# DAILY ASSOCIATIONS OF ACTIGRAPHIC SLEEP AND NEXT-DAY WORKING MEMORY IN MIDLIFE AND OLDER ADULTS

**DOI:** 10.1093/geroni/igae098.0246

**Published:** 2024-12-31

**Authors:** Lindsay Master, Orfeu Buxton, Alexa Allan, Courtney Fine, Jacqueline Mogle, Alan Zonderman, Michele Evans, Alyssa Gamaldo

**Affiliations:** The Pennsylvania State University, University Park, Pennsylvania, United States; The Pennsylvania State University, University Park, Pennsylvania, United States; The Pennsylvania State University, University Park, Pennsylvania, United States; Clemson University, Clemson, South Carolina, United States; Clemson University, Clemson, South Carolina, United States; National Institute on Aging, Baltimore, Maryland, United States; National Institute on Aging, Baltimore, Maryland, United States; Clemson University, Clemson, South Carolina, United States

## Abstract

This study evaluates the day-to-day associations between actigraphic nighttime sleep and next day working memory in a sample of diverse midlife and older adults. The study used a subsample of the HANDLSleep Study which provided complete information of the variables of interest. Preliminary data from wave 5 (2022-current, n=104, 72% female, 55% Black adults, mean age: 62) were collected via a wrist-worn accelerometer and daily mobile phone cognitive tasks over one week, from which nighttime sleep measures and next-day working memory outcomes were derived. Multilevel linear models tested within-person associations of nightly sleep duration, timing, and quality with next-day mean reaction time calculated from the Multiple Object Tracking (MOT) Task via a mobile device, which evaluates working memory. Analyses were stratified by poverty status (above/below 125% federal poverty line) and adjusted for age, sex, race, WRAT3 literacy scores, average sleep measures, and weekend/weekday. Within individuals who live below the poverty line (n=47), on nights when participants fell asleep earlier (p=.013) and slept longer (p=.021) but had more minutes of wake (p=.0013) and less efficient sleep (p=.018) than their average, next-day mean reaction time was faster. Sleep measures were not associated with next-day working memory for individuals who live equal/above the poverty line. Our results demonstrate that within-person improvements to nightly sleep timing and duration may help next-day reaction time on working memory in adults who live below the poverty line.

